# Silver Nanoclusters with Broad-Spectrum Antibacterial Properties

**DOI:** 10.1021/acsomega.5c00163

**Published:** 2025-06-17

**Authors:** India J. Cook, Maria Eleni Kyriazi, Myron Christodoulides, Antonios G. Kanaras

**Affiliations:** † Physics and Astronomy, Faculty of Physical Sciences and Engineering, 7423 University of Southampton, Southampton SO17 1BJ, U.K.; ‡ College of Engineering and Technology, American University of the Middle East, Egaila 15453, Kuwait; § Neisseria Research Laboratory, Molecular Microbiology, School of Clinical and Experimental Sciences, Sir Henry Welcome Laboratories, Faculty of Medicine, University of Southampton, Southampton SO16 6YD, U.K.; ∥ Institute for Life Sciences, University of Southampton, Southampton SO17 1BJ, United Kingdom; ⊥ Department of Chemistry, National and Kapodistrian University of Athens, Athens 15771, Greece

## Abstract

Bacterial resistance to antibiotics is a significant threat to global health, and innovative strategies are needed to tackle these bacterial infections. In this study, we used an in vitro bactericidal assay to demonstrate that small silver nanoclusters (AgNCs) coated with 5-mercapto-2-nitrobenzoic acid (MNBA) exhibit bactericidal activity against a range of Gram-negative and Gram-positive ESKAPEE bacteria and bacteria belonging to the WHO Priority Pathogen List. Understanding the broad-spectrum antibacterial properties of MNBA-AgNCs highlights the importance of nanoparticle customization in further combating antimicrobial resistance.

## Graphical abstract

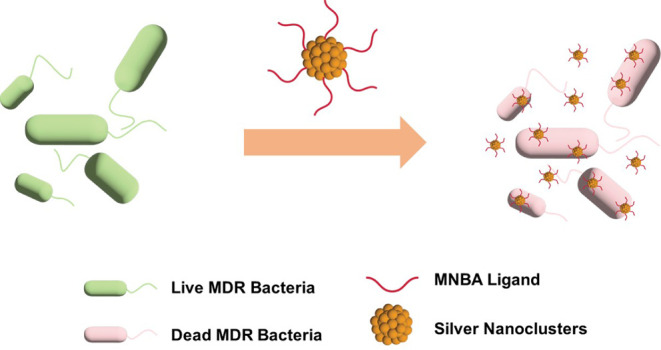

## Introduction

Antimicrobial resistance (AMR) is recognized by the World Health Organization (WHO) as one of the top challenges to public health.^1,2^ Most clinically approved antibiotics, currently used to treat bacterial infections, are derived from natural products sourced from fungi, plants, soil, or microorganisms.^3^ However, through coevolution, bacteria have evolved mechanisms to subvert antibiotic killing, and the emergence of resistance is considered an inevitable consequence of the widespread use of antibiotics.^4^ There are multiple ways that resistance is achieved, which include restriction of drug absorption, drug inactivation, target protection or modification, and active drug efflux.^5^ The chosen mechanism of resistance is influenced by the type of drug, the local microenvironment, and the structure of the bacteria itself.^6^ The Gram-negative bacterial cell wall consists of an outer membrane (OM), a 2–3 nm thin peptidoglycan (PG) layer, and an inner membrane (IM). The OM is composed of a lipid bilayer that contains a variety of proteins and negatively charged lipopolysaccharides, and acts as a physical barrier against the surrounding environment.^7^ By contrast, the Gram-positive cell wall consists of a 20–80 nm thick PG layer, up to 90%, teichoic acids, and lipoids.^8^

Given the rise in AMR, there is a need for new antimicrobial compounds to add to the arsenal of antibiotics currently used as adjuncts. In recent years, the field of nanotechnology has been investigated as a promising avenue for creating customized nanoparticles that can effectively contribute to the killing of bacteria. For example, significant advancements have been made in the synthesis of antibacterial metal nanoparticles with various chemical compositions, including zinc, copper, gold, and silver.^9–12^ Such nanoparticles can be further customized via manipulation of their size, morphology, and surface chemistry to enhance biological properties, including bactericidal activity, biocompatibility, and reduced cytotoxicity.^12–18^ In particular, silver-based nanoparticles (AgNPs) have emerged as promising candidates for combating bacterial infections and AMR due to their superior potency and biocompatibility.^9,19^ The antimicrobial properties of silver-containing compounds are actively researched and, in several cases, have led to the development of commercial products such as antibacterial wound dressings.^20,21^

For example, Khalifa et al. used an agar well-diffusion assay to demonstrate that AgNPs (33 to 90 nm) produced by probiotic bacteria exhibited antibacterial efficacy comparable to traditional antibiotics against four multidrug-resistant pathogenic bacteria (*Proteus vulgaris*, *Escherichia coli*, *Staphylococcus aureus*,*and* *Klebsiella pneumoniae*), and were not toxic to treated rats.^21^ Rahman et al. similarly used an agar well-diffusion assay to demonstrate the dose-dependent antibacterial activity of surfactin (SUR)-coated AgNPs (22 nm) against a multidrug-resistant strain of *Pseudomonas aeruginosa*.^22^ Furthermore, they investigated the antiadherent and antibiofilm properties of SUR-AgNPs and showed that the formation of biofilm rings increased as NP concentration decreased. Additionally, increasing the concentration of SUR-AgNPs tested on preformed biofilms resulted in increased activity against the biofilms.^1^ The antibacterial and antiadhesive properties of Ag-based nanoparticles were also investigated by Khalid et al., who showed that rhamnolipid-coated AgNPs (35 nm) demonstrated antibiofilm and antiadhesive properties against *P. aeruginosa* and *S. aureus*.^23^

One important property that influences the bactericidal effectiveness of nanoparticles (NPs) is their size. While a direct comparison of silver nanoparticles of different sizes is not straightforward because of the different surface ligands used and the different density of surface ligands, which synergistically affect the bactericidal efficacy, there are several studies that show smaller silver nanoparticles are significantly more reactive than larger ones and more effective in killing bacteria.^24–26^ As a result, the antibacterial activity of metal NPs consisting of a few atoms, typically less than 2 nm in diameter, also known as nanoclusters (NCs), is actively researched.^18^ Their increased antibacterial activity is due to their significantly larger surface area, higher reactivity of surface atoms, and enhanced interaction with biomolecules of similar size.^27,28^ NCs composed of a silver metal core (AgNCs) show more prominent antibacterial properties due to the high reactivity of silver surface atoms.^29^

Here, we demonstrate that ultrasmall silver nanoclusters functionalized with 5-mercapto-2-nitrobenzoic acid (MNBA), a negatively charged and small aromatic ligand, are highly effective against a wide range of bacteria recognized as WHO high-priority pathogens for the development of new antimicrobials. MNBA-AgNCs were highly efficient in killing critical bacteria that exhibit increased resistance to common antibiotics and may open new avenues for broader chemical customization and applicability of this family of NCs.

## Results and Discussion

### Synthesis and Characterization of MNBA-AgNCs

Silver nanoclusters were synthesized and functionalized as previously reported.^30–33^ The synthesis involved the reduction of a soluble silver precursor in the presence of MNBA ligands. The MNBA ligand is an organic compound belonging to the class of nitrobenzoic acids. It consists of a benzene ring with a nitro group (−NO_2_), a carboxylic acid group (−COOH), and a mercapto group (−SH). These groups are found at positions 2, 1, and 5, respectively, on the benzene ring. The nitro groups are relatively hydroneutral, and the carboxylic acid groups are hydrophilic, thus facilitating their solubility within aqueous media. MNBA strongly binds to the silver surface through the thiol group, resulting in atomically monodisperse nanoclusters.^31^

Silver NCs were characterized using UV–visible spectroscopy, where four well-defined absorption peaks at ∼400, ∼480, ∼550, and ∼650 nm were observed, confirming the molecular-like electronic structure of AgNCs (Figure S1).^31^ These peaks are characteristic of AgNCs and result from the contribution of many factors related to the nanocluster size, specific shape, and ligand coating.^34,35^ Furthermore, transmission electron microscopy (TEM) verified a narrow size distribution (Figure S2A). A size-dependent histogram was plotted by measuring the diameter of ∼350 nanoclusters, and the mean size of AgNCs was determined to be ∼1.8 nm (Figure S2B).^36^

### Bactericidal Efficacy of AgNC-MNBAs

We tested MNBA-AgNCs against a variety of Gram-negative and Gram-positive bacteria, including examples of the ESKAPEE pathogens (i.e., *Enterococcus faecium*, *Enterococcus faecalis*, *Staphylococcus aureus*, *Klebsiella pneumoniae*, *Acinetobacter baumannii*, *Pseudomonas aeruginosa*, *Enterobacter* species, and *Escherichia coli*), organisms listed in the WHO Priority Pathogen list (*Haemophilus influenzae*, *Streptococcus agalactiae* (*Group B* *Streptococcus* *, GBS*), *S. pyogenes* (*Group A* *Streptococcus*, *GAS*), and some that can cause human and animal infections (*Streptococcus suis*).^37^ Multidrug-resistant ESKAPEE pathogens share common attributes, particularly the ability to cause hospital-acquired infections that may affect all major organs. GBS causes neonatal meningitis, and GAS causes multimorbidities such as necrotizing fasciitis, toxic shock syndrome, pneumonia, and among others. *S. suis* is a zoonotic organism causing sepsis and meningitis in pigs and humans.^38,39^ *H. influenzae* type B is a classical agent of meningitis, septicemia, and otitis media in children.^40^ Gram-negative and Gram-positive bacteria have unique structural differences that are fundamental to understanding their behavior and response to external environmental conditions, which include the presence of antibiotics or NPs.

#### Silver Nanoclusters against Gram-Negative Bacteria

Gram-negative organisms belonging to the ESKAPEE family and present in the WHO Priority Pathogens List – *P. aeruginosa*, *A. baumannii*, *K. pneumoniae*, and *E. coli* *–* and *H. influenzae* were treated for 1 h with various doses of MNBA-AgNCs using a standard minimum bactericidal concentration (MBC) assay (Figure 1). All the Gram-negative bacteria were susceptible to the MNBA-AgNCs, and the calculated MBC50 and MBC > 90 values are reported in Table S1. The most susceptible organisms were *A. baumannii* and *H. influenzae*, with recorded MBC50 and MBC > 90 values of 0.056 and 0.24 μM for the former and MBC50/MBC > 90 of 0.09–0.24 μM for the latter. By contrast, higher doses were needed to kill *P. aeruginosa*, *E. coli*, and *K. pneumoniae*, with MBC50 values of 0.45, 1.75, and 0.22 μM, and MBC > 90 values of 2.14, 9.52, and 8.21 μM for the three pathogens, respectively (Table S1). Increased MBC > 90 values were consistent with the need for higher doses of the compound to kill >90% of the bacterial loads in the experiments. To confirm that the MNBA-AgNCs were toxic only to bacteria and not to eukaryotic cells, we tested the viability of human Chang conjunctival cells against varying concentrations of MNBA-AgNCs (0.028–58.44 μM). No significant cytotoxicity was observed following treatment of the cells for 18 h with the MNBA-AgNCs, which showed that the particles exhibited a targeted effect on bacteria while remaining nontoxic to human cells, thus highlighting their specificity and safety as previously reported (Figure S3).^36^ All of the bacteria were sensitive to treatment with antibiotics, which were used as internal controls in the experiments (Figure S4 and Table S2). However, the disadvantage of the antibiotics is the capability of all these organisms to easily develop resistance, thus requiring increasing antibiotic MICs. By contrast, the development of resistance of bacteria to MNBA-AgNCs is more challenging due to the multiple ways the nanoclusters can kill the bacteria. These included other ESKAPEE organisms, i.e., the Gram-positive *Enterococcus faecalis*, *Staphylococcus aureus*, and others on the WHO Priority Pathogen List, i.e., GBS, GAS, and *S. suis*.

**Figure 1:**
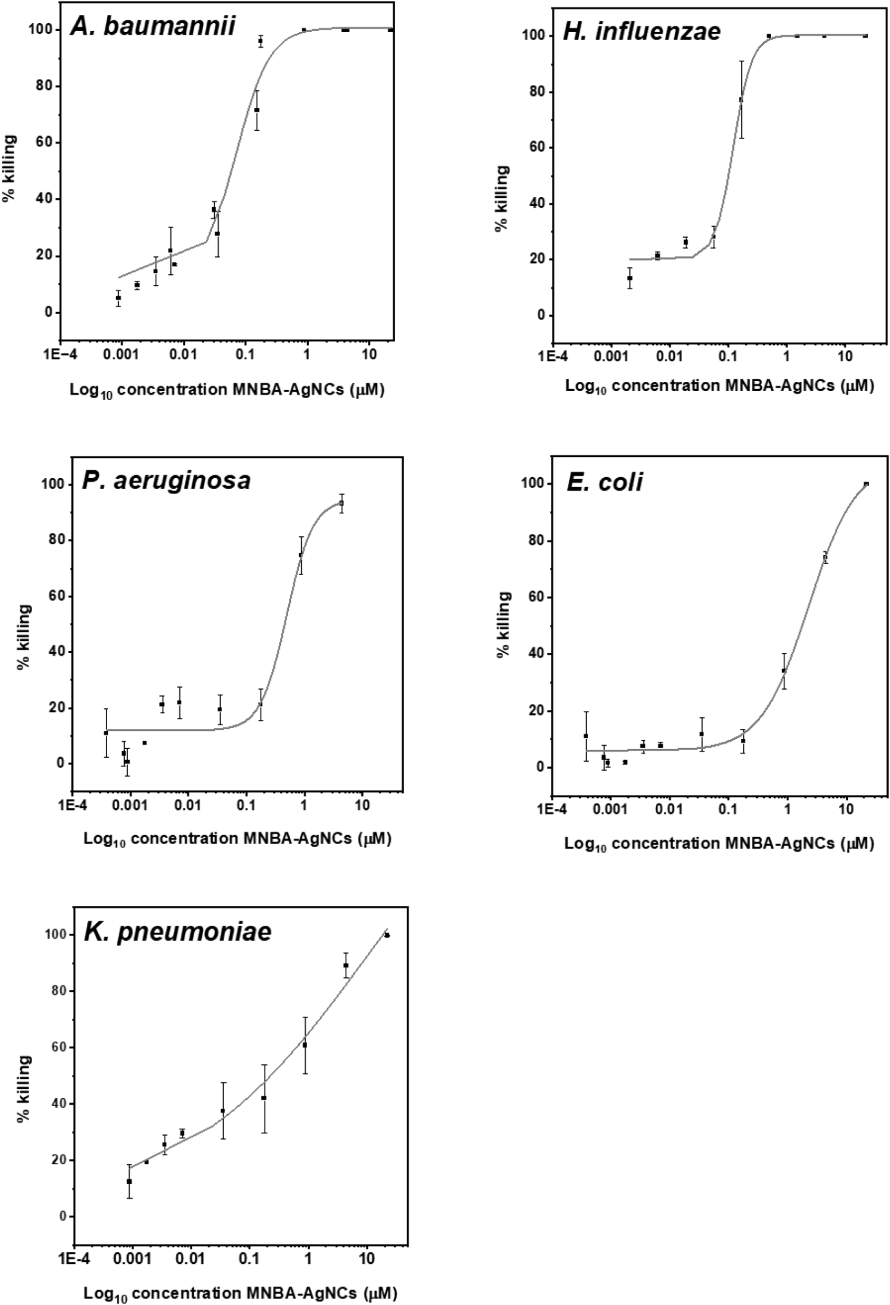
Percentage killing of Gram-negative bacteria following a 1 h incubation with increasing concentrations of MNBA-AgNCs. The symbols represent the mean, and the error bars represent the standard error of the means of at least *N* = 3 experiments.

Examination of the titration curves showed that some of the Gram-positive bacteria were sensitive to the MNBA-AgNCs and others were more difficult to kill. The most sensitive were *E. faecium*, with MBC50 and MBC > 90 values of 0.14 and 0.17 μM, respectively, and *S. suis*, with MBC50 and MBC > 90 values of 0.17 and 0.67 μM, respectively (Table S1). Increasing doses were needed to kill GBS (MBC50 and MBC > 90 of 0.55 and 3.54 μM) and GAS (MBC50 and MBC > 90 of 0.86 and 9.21 μM). Higher doses of MNBA-AgNCs were needed to kill *S. aureus*, with a recorded MBC50 value of 7 μM (Figure 2 and Table S1) and killing did not reach a 90% value even at the highest dose of 22 μM tested. By contrast, not even the highest dose of 22 μM could produce 50% killing of *E. faecalis*.

**Figure 2:**
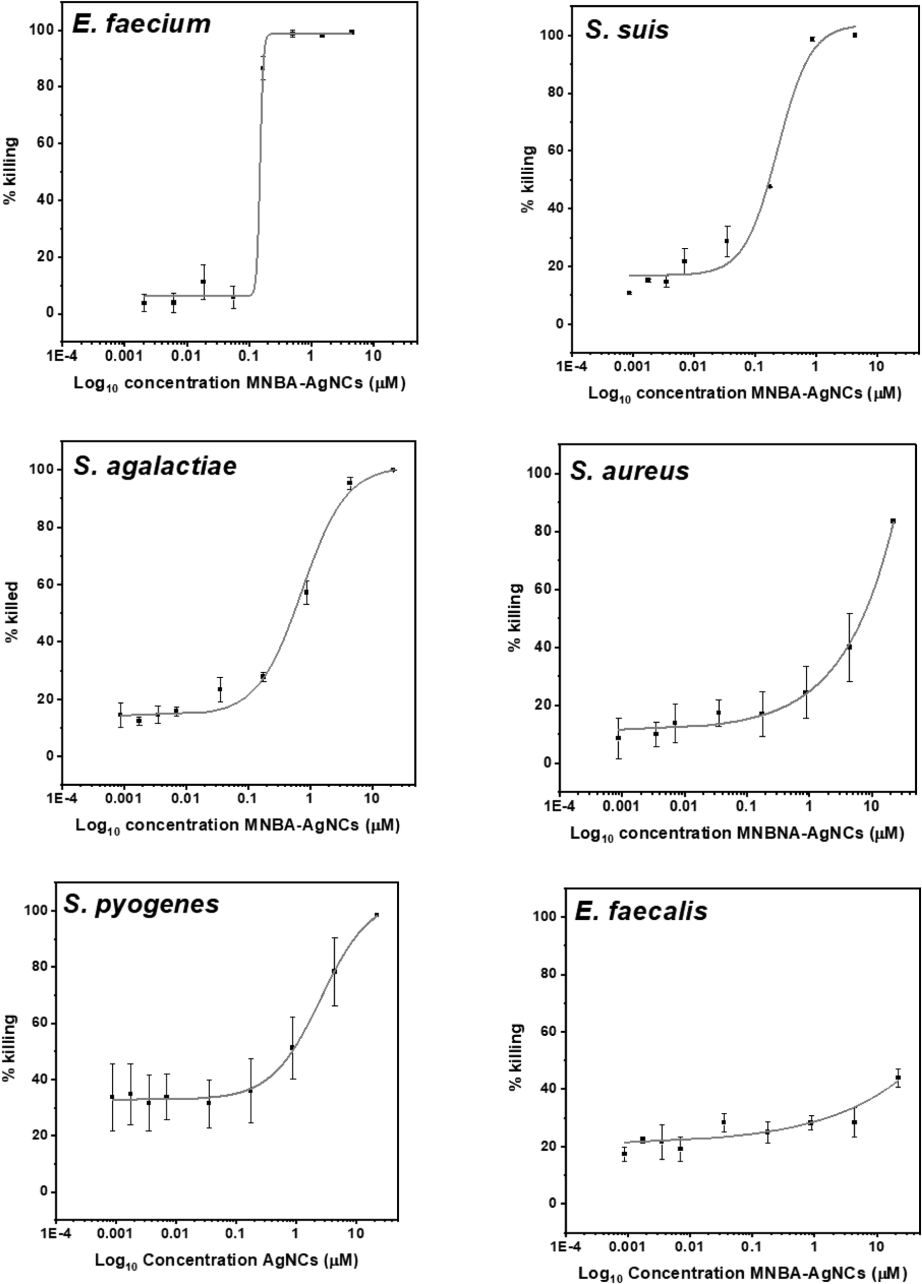
Percentage killing of Gram-positive bacteria following a 1 h incubation with increasing concentrations of MNBA-AgNCs. The symbols represent the mean, and the error bars represent the standard error of the means of at least *N* = 3 experiments.

To determine whether the viability of *E. faecalis* was not strain-specific, we also tested the MNBA-AgNCs against four additional clinical isolates collected from patients with sepsis (Figure 3). No significant bactericidal activity was observed against any of the strains tested up to 22 μM of MNBA-AgNCs. Several hypotheses can be raised to explain the lack of activity against *E. faecalis* when compared to that against the other Gram-positive organisms. The basic Gram-positive cell membrane consists of a PG backbone (90% of total cell wall weight), anionic polymers (teichoic acids and cell wall polysaccharides), and cell wall-associated and wall-anchored proteins.^41^ There are distinct differences in the cell wall structures and cell wall-associated and wall-anchored proteins of the different Gram-positive bacteria tested in our study,^42–47^ many of which play important roles in virulence and may be involved in mediating resistance to antimicrobials. Such differences in the cell wall structure may impact the ability of MNBA-AgNCs to exert their bactericidal effect on *E. faecalis*, though the exact mechanism remains to be identified. One study reported that green-synthesized Ag-NPs could combat multidrug-resistant *E. faecalis* via reducing virulence factors.^48^ It is also possible that MNBA-AgNCs are unable to penetrate the *E. faecalis* cell wall efficiently, or *E. faecalis* resistance could be realized by triggering silver nanoparticle surface aggregation, as has been reported for *S. aureus*.^49^

**Figure 3:**
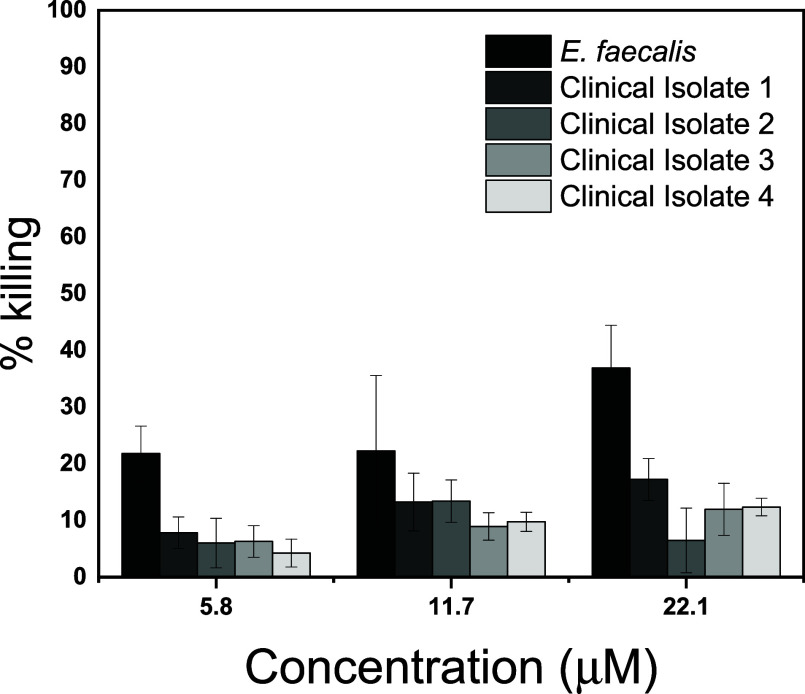
Activity of MNBA-AgNCs against different *E. faecalis* isolates. A selection of enterococcal isolates from patients with sepsis were treated with various doses of MNBA-AgNCs (μM), and bacterial viability was measured using a standard MBC assay. The columns represent the mean, and the error bars represent the standard error of the means of at least *N* = 3 experiments.

Beyond the potential global mechanisms of resistance related to the cell wall, several studies have examined resistance to AgNPs at the gene expression and proteome levels. For example, it has been reported that *E. faecalis* can build resistance to AgNPs^50^ and proteomics analyses showed that resistance consisted of the expression of many proteins that appeared to be involved in counteracting the antimicrobial mechanisms of the AgNPs. These included proteins associated with metabolic processes, transport, cell redox homeostasis, cellular ion homeostasis, stress response, and DNA/RNA repair mechanisms.^50^ *E. faecalis* colonies were also established with strong resistance against both Ag^+^ ions and AgNPs.^51^ Following genome sequencing and analyses of resistant bacteria compared with sensitive bacteria, most of the upregulated genes were related to the tricarboxylic acid cycle, suggesting that the Ag^+^-resistant ability was related to the enhancement of cellular energy metabolism. It was suggested that the Ag^+^-resistant ability of *E. faecalis* was due to enhanced Ag^+^-efflux activity that was boosted by the enhanced cellular energy metabolism.^51^ Other upregulated gene expressions for Ag^+^/AgNPs were observed for sulfur compound metabolism, protein homo-oligomerization, metabolic capacity, purine nucleotide metabolism, and N-acetylsalicylic acid metabolism. Speculatively, sulfur compound metabolism may be beneficial for *E. faecalis* to chelate AgNPs by sulfhydryl groups and detoxify AgNPs; further detoxification may be achieved by protein homo-oligomerization to produce functional multisubunit proteins; and enhanced metabolic activity may favor the growth and development of *E. faecalis* bacteria in the silver environment. A different possibility is that Lipid II-interacting antibiotic response regulator (LiaR)-independent pathways are functioning in *E. faecalis* ^52^ to regulate cell wall adaptation in response to antibiotics, e.g., both to ceftriaxone used in our study and to the MNBA-AgNCs.

It has been reported that the antimicrobial efficiency of silver particles is higher against Gram-negative bacteria than Gram-positive bacteria.^53^ The cell wall of Gram-negative bacteria has a stronger negative charge than that of Gram-positive bacteria due to the presence of lipopolysaccharides. This, in turn, was suggested to promote adhesion of some types of NPs, causing the bacteria to be more susceptible to the NP antimicrobial action.^54^ Furthermore, it has been discussed that the uptake of certain types of AgNPs is higher in Gram-negative bacteria, and the peptidoglycan-thick cell wall of Gram-positive bacteria could serve as a barrier against the permeation of Ag^+^ ions. Moreover, Ag^+^ ions have been shown to penetrate the cytoplasm of Gram-negative bacteria more easily, which results in cell lysis.^55^

Due to their small size, it is possible that MNBA-AgNCs interact effectively with the different membranes of bacteria, and in many cases, they could cause significant membrane disruption and rupture.^56^ Chen and coworkers used AgNCs with amphiphilic ligands, which showed strong interactions with bacterial cells, leading to efficient endocytosis and increased killing efficacy in vitro against *P. aeruginosa*. Furthermore, when compared to AgNPs (30 and 50 nm) a significant difference in bactericidal efficacy was reported, thus demonstrating the importance of size.^25^ Future studies could examine whether susceptibility is influenced by selective factors such as membrane composition, e.g., the ratios of teichoic acids and peptidoglycans in Gram-positive organisms, different outer membrane protein compositions in Gram-negative organisms, and the presence or absence of efflux pumps. Our MBC bactericidal assay, wherein the bacteria were treated with MNBA-AgNCs, would suggest a hypothesis that rapid cidal effects were likely due to the disruption of bacterial membranes.^36^

## Conclusions

In conclusion, MNBA-AgNCs were tested for their efficacy against a broader range of Gram-negative and Gram-positive bacteria, including WHO Priority Pathogens. Our data demonstrated the dose-dependent efficacy of MNBA-AgNCs against most bacteria treated, with the exception of *S. aureus* and *E. faecalis*. While there was variability in the MBC values among different bacteria, the consistent bactericidal activity observed supports the broad-spectrum antimicrobial activity and potential versatility of the MNBA-AgNCs. These small silver nanoclusters could serve as a promising therapeutic approach adjunctive to antibiotic treatment, for example, to treat skin infections by incorporating them into wound dressings and as coatings for medical devices and bandages. Moreover, due to their broad-spectrum antibacterial activity, they could also be used as surface disinfectants, especially in hospitals, effectively addressing the growing challenge of antibiotic resistance.

## Experimental Procedures

### Reagents and Chemicals

5,5′-Dithio-bis­(2-nitrobenzoic acid) (DTNBA), sodium hydroxide, silver nitrate, sodium borohydride (NABH_4_) and methanol (99%) were purchased from Thermo Fisher Scientific. Media used for bacterial culture were purchased from Sigma.

### Synthesis and Characterization of Silver Nanoclusters

Colloidal silver nanoclusters were synthesized following an established protocol.^31,36^ Briefly, a solution of DTNBA (9.9 mg, 25 mmol) was dissolved in a solution of sodium hydroxide (20 mL, 1 M) and stirred at room temperature for 45 min. Subsequently, an aqueous solution of silver nitrate (5 mL, 10 mM) was added, and the mixture was stirred vigorously for an additional 45 min. The formation of silver-TNB complexes was evident from the change in color from dark yellow to greenish-yellow. Finally, a freshly prepared ice-cold solution of NABH_4_ (2 mL, 13.2 mM) was added, which quickly turned the solution dark burgundy, indicating the formation of silver nanoclusters. The reaction was stirred for a further 4 h, enabling the formation of NCs. The final product was then purified via repeated precipitation using a 50% (v/v) methanol solution, followed by storage overnight at −5 °C and centrifugation at 7000 rpm. This step was repeated until the supernatant was nearly colorless. The optical properties of the nanoclusters were assessed by using UV–visible spectroscopy. TEM images were taken on a Hitachi HT7700 transmission electron microscope and analyzed using ImageJ software (National Institutes of Health, USA).

### Bacteria

*Acinetobacter baumannii* ATCC19606, *Enterococcus faecalis* ATCC29212 and *Enterococcus faecium* strain NCTC 7171 (ATCC-19434) were all obtained from LGC Standards, Teddington, UK. *Pseudomonas aeruginosa* strain PAO1 (Holloway 1C Stanier 131) was obtained from the National Collection of Industrial, Food, and Marine Bacteria, UK. *Haemophilus influenzae* type B strain Eagan was isolated from the cerebrospinal fluid of a patient presenting with meningitis in Boston, USA (Anderson et al., 1972), and was obtained from the Department of Pediatrics, John Radcliffe Hospital, Oxford, UK. *Streptococcus agalactiae* strain 2603 V/R was obtained from ATCC (BAA611). *Staphylococcus aureus* NCTC 8325-4, *Streptococcus pyogenes* strain NCTC 8191, and *Klebsiella pneumoniae* NCTC 9634 were obtained from the National Collection of Type Cultures, Porton Down, Salisbury, UK. *Streptococcus suis* strain SC84 has been described previously.^57^ *Escherichia coli* DSM (018:K1:H−) is a spontaneous nalidixic acid-resistant strain of *E. coli* RS228, which was originally isolated from a fecal specimen from a healthy individual.^58^ This strain was shown to be pathogenic in the infant rat model of bacteremia and meningitis.^59^

*A. baumannii*, *P. aeruginosa*, *S. aureus*, and *Enterococcus* spp. were all grown on nutrient agar; all streptococci were grown on Brain Heart Infusion agar; *H. influenzae* was grown on *Haemophilus* test medium (HTM) supplemented with 15 μg/mL of β-nicotinamide adenine dinucleotide (β-NAD) and hematin; *E. coli* was grown on Luria–Bertani agar; and *K. pneumoniae* was grown on trypticase soy agar. All media were obtained from Oxoid, Basingstoke, UK.

### Bactericidal Assay

The bactericidal potency of MNBA-AgNCs was quantified using a Minimum Bactericidal Concentration (MBC) assay as described previously.^36,60^ Appropriate agar plates were inoculated with bacteria directly from frozen stocks and cultured at 37 °C with 5% (v/v) CO_2_ for 16 h. Logarithmic growth phase subcultures were then prepared by transferring a small quantity of the overnight culture to a fresh agar plate and incubating for a further 6 h. Master bacterial suspensions were then prepared by suspending bacteria into phosphate-buffered saline (PBS), and the absorbances of SDS/NaOH-lysed aliquots were measured with a Nanodrop at λ = 260 nm to estimate the bacterial concentration in the master solutions, and final suspensions were made of 1.25 × 10^4^ colony forming units (CFU)/mL.

To triplicate wells of a sterile 96-well plate (Greiner, Merck, Gillingham, UK), 80 μL of bacterial suspension (∼1000 CFU) and 20 μL of MNBA-AgNCs at various concentrations, obtained through serial dilution in PBS, were added. Controls included bacteria alone and bacteria treated with antibiotics specific to each organism. The plate was incubated for 1 h at 37 °C with 5% (v/v) CO_2_, after which 15 μL aliquots from each well were plated onto fresh agar plates. Agar plates were incubated at 37 °C with 5% (v/v) CO_2_ for 24 h, and surviving bacterial colonies were counted on a ProtoCOL colony counter (Synoptics, Cambridge, UK).

The percentage of bacteria killed by MNBA-AgNCs during the 1 h incubation period was determined in comparison to the negative control (bacteria alone) as follows: (CFU/mL in treated sample positive control – CFU/mL in positive control after incubation)/CFU/mL in positive control. Concentration-dependent bactericidal efficacy was determined by fitting a regression curve to the data points to determine the MBC50 and MBC90 values, representing the concentrations of AgNCs required to achieve 50% and 90% reduction of bacterial colonies in 1 h. The assay was performed at least 4 times for each species of bacteria.

#### Assessing the Cytotoxicity of MNBA-AgNCs

Cytotoxicity was determined as described recently.^61^ Human Chang conjunctival epithelial cells (European Type Culture Collection, Porton Down, United Kingdom) were cultured in the wells of sterile 96-well cell culture plates (Nunc) at 37 °C in Dulbecco’s modified Eagle’s medium supplemented with Glutamax-1 and sodium pyruvate (DMEM) (Lonza, United Kingdom) and 10% (v/v) decomplemented fetal calf serum (dFCS) (Lonza). Cells were cultured in a humidified atmosphere at 37 °C with 5% (v/v) CO_2_. Prior to treatment with MNBA-AgNCs, the medium was removed, and the cells were washed to remove any dead cells, and fresh medium was added (180 μL/well). Next, 20 μL of MNBA-AgNCs was added per well, with a final starting concentration of 58.44 μM in the well. A 2-fold dilution series was tested in triplicate wells. Lysis solution (1% (w/v) sodium dodecyl sulfate in 0.1 M NaOH) was added as a positive control. Negative controls were cells alone without treatment. The plates were incubated for 18 h at 37 °C with 5% (v/v) CO_2_, and then 20 μL of resazurin (Merck, United Kingdom) was added to each well. The plate was incubated for a further 6 and 18 h, and the absorbance was read at λ = 570 and λ = 595 nm for background correction on a SpectraMax iD3 plate reader. Cytotoxicity was calculated as described previously.^62^

## Supplementary Material



## Notes

Detailed characterization of the silver nanoclusters (UV–vis absorption spectrum, TEM of MNBA-AgNCs, etc.); a table summarizing the MBC values of the interactions between bacteria and silver nanoclusters; titration curves; a table summarizing the interactions of the bacteria with antibiotics (PDF). The raw data is available at https://doi.org/10.5258/SOTON/D3453.
