# Supplementary material for: Silver Nanoclusters with Broad-Spectrum Antibacterial Properties
Source: ACS Omega. 2025 Jun 17;10(25):26520–8. doi: 10.1021/acsomega.5c00163 (PMC12224105; doi:10.1021/acsomega.5c00163)
Supplement: Supplementary file 1 [file ao5c00163_si_001.pdf]

Supporting Information for

Silver Nanoclusters with Broad Spectrum Antibacterial Properties

India J. Cook,[a] *Maria Eleni Kyriazi,*[b] *Myron Christodoulides*[c, d,]* *and Antonios G. Kanaras*[a, d,e,]*

[a] Physics and Astronomy, Faculty of Physical Sciences and Engineering, University of Southampton, Southampton, SO17 1BJ, United Kingdom.

[b] College of Engineering and Technology, American University of the Middle East, 54200, Egaila, Kuwait.

[c] Neisseria Research Laboratory, Molecular Microbiology, School of Clinical and Experimental Sciences, Sir Henry Welcome Laboratories, Faculty of Medicine, University of Southampton, Southampton, SO16 6YD, United Kingdom.

[d] Institute for Life Sciences, University of Southampton, Southampton SO17 1BJ, United Kingdom.

[e] Department of Chemistry, National and Kapodistrian University of Athens, 15771, Athens, Greece.

Table of contents

a. **UV-Vis spectroscopy** **S-3**

b. **Transmission electron microscopy** **S-4**

c. **Minimum Bactericidal Concentrations for MNBA-AgNCs against different bacteria.** **S-5**

d. **Cytotoxicity of MNBA-AgNCs upon incubation with human cells** **S-6**

e. **Titration curves for antibiotics tested against the different bacteria** **S-7**

a. UV-Vis spectroscopy

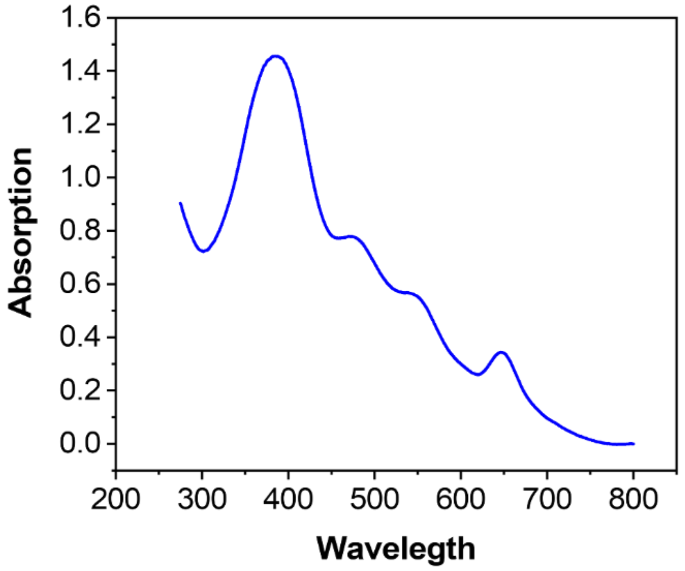

Figure S1. UV absorption spectrum of MNBA-AgNCs. The spectrum shows four peaks at ~400, ~480, ~550 and ~650 nm characteristic for silver nanoclusters.

b. Transmission electron microscopy

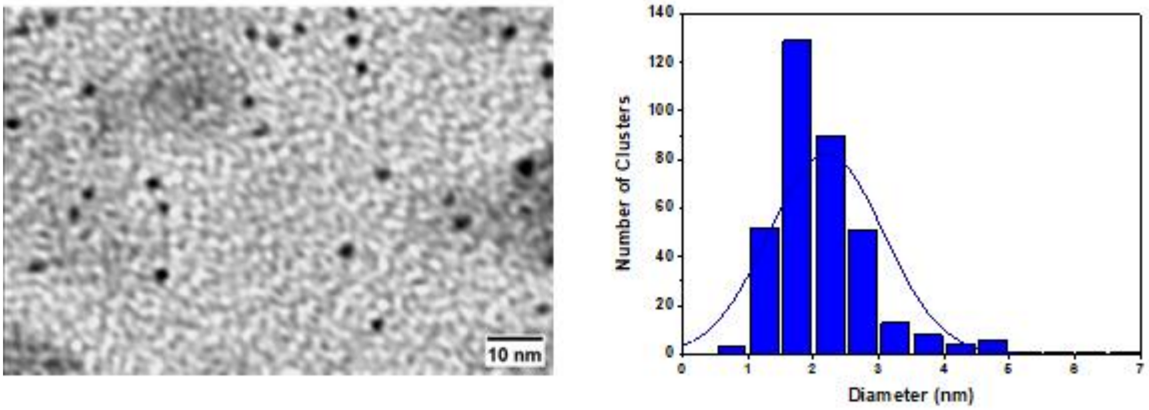

Figure S2. Representative TEM image of MNBA-AgNCs (scale bar is 10 nm) with corresponding size distribution histogram. The MNBA-AgNCs were found to have an average size of 1.8 ± 0.8 nm.

c. **Minimum Bactericidal Concentrations for MNBA-AgNCs against different bacteria.**

Table S1. MBC 50 and MBC>90 values for bacteria treated with MNBA-AgNCs

**Bacteria**		**ESKAPEE**	**WHO Priority**	**MBC50 (µM)**	**MBC>90 (µM)**
Gram-negative	*E. coli*	Yes	1: Critical	1.75	9.52
	K. pneumoniae	Yes	1: Critical	0.22	8.21
	A. baumannii	Yes	1: Critical	0.056	0.24
	P. aeruginosa	Yes	2: High	0.45	2.14
	H. influenzae	No	3: Medium	0.99	0.24
Gram-positive	*E. faecium*	Yes	2: High	0.14	0.17
	E. faecalis	Yes	2: High	>22	>22
	S. aureus	Yes	2: High	7.04	>22
	S. suis	No	-	0.17	0.67
	S. pyogenes	No	3: Medium	0.86	9.21
	S. agalactiae	No	3: Medium	0.55	3.54

d. Cytotoxicity of MNBA-AgNCs for human cells *in vitro*

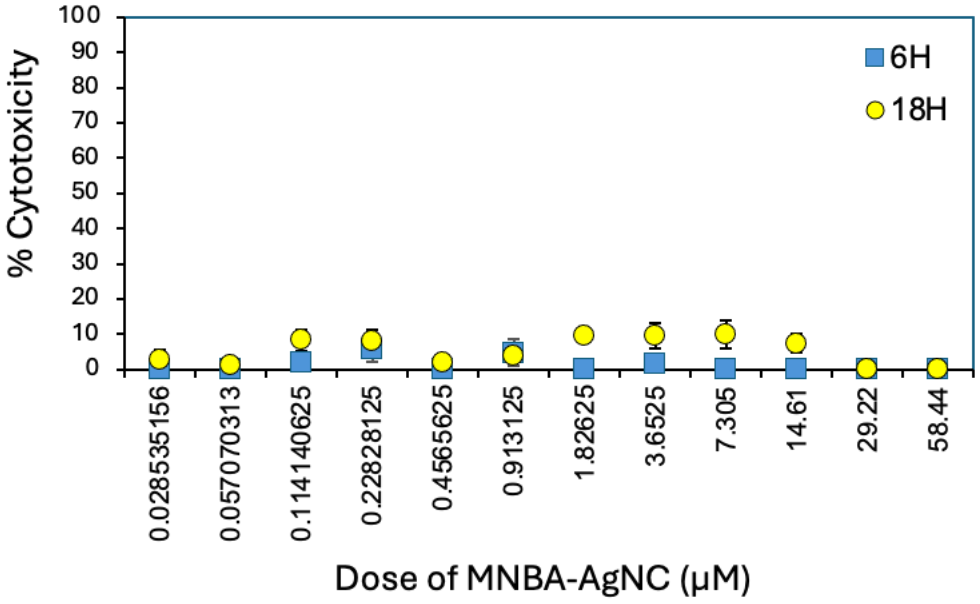

Figure S3. Human Chang conjunctival cells were treated with various doses of MNBA-AgNCs in triplicate for 18h and viability was assessed with a resazurin assay at 6 and 18h. The symbols represent the mean % cytotoxicity from n=4 independent experiments and the error bars the standard errors of the means.

e. Titration curves for antibiotics tested against the different bacteria

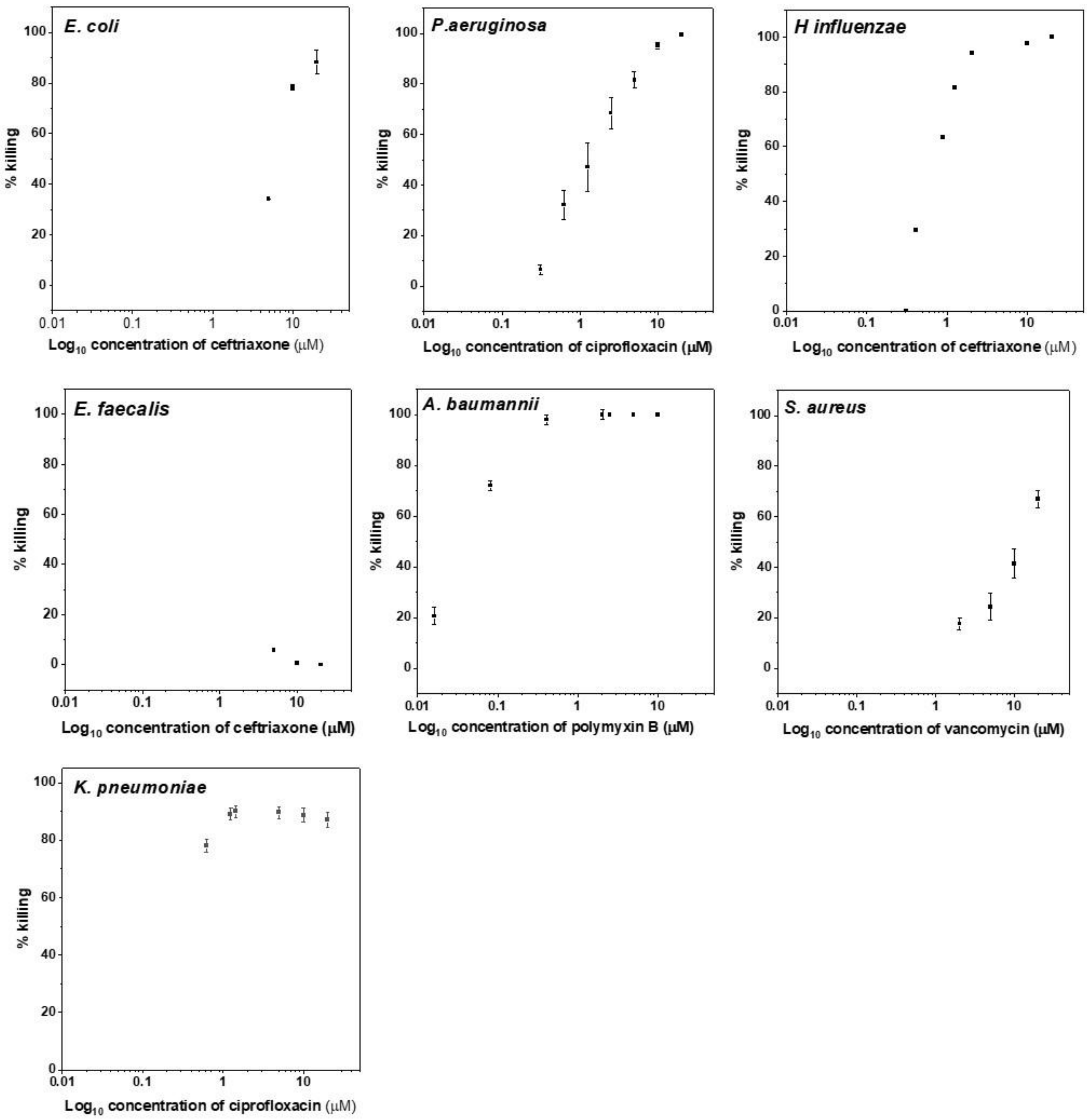

Figure S4. Percentage killing of bacterial strains against increasing log concentrations of antibiotics. *E. coli, H. influenza* and *E. faecalis* were treated with ceftriaxone, *P. aeruginosa* and *K. pneumonia* were treated with ciprofloxacin, *A. baumannii* was treated with polymixin B and finally *S. aureus* was treated with vancomycin

Table S2. MBC values for antibiotics tested against the different bacteria.

Bacteria		MBC50 (µM)	MBC>90 (µM)
Gram-negative	*E. coli*	5	11
	K. pneumoniae	NA	2
	A. baumannii	0.04	0.2
	P. aeruginosa	1.8	7
	H. influenzae	0.9	1.5
Gram-positive	*E. faecalis*	>11	>11
	S. aureus	11	>11
